# Physical function in older people with rheumatoid arthritis and population controls: a cross-sectional study of self-reported and performance-based measures

**DOI:** 10.1007/s00296-026-06204-2

**Published:** 2026-06-17

**Authors:** Saskia P. M. Truijen, Annelies Boonen, Sofia Ramiro, Marloes van Onna

**Affiliations:** 1 Department of Rheumatology, Maastricht University Medical Center+, P. Debyelaan 25, Maastricht, 6229 HX The Netherlands; 2 Care and Public Health Research Institute (CAPHRI), Maastricht University, Minderbroedersberg 4-6, Maastricht, 6211 LK The Netherlands; 3 Department of Rheumatology, Leiden University Medical Center, Albinusdreef 2, Leiden, 2333 ZG The Netherlands; 4 Department of Rheumatology, Zuyderland Medical Center, Henri Dunantstraat 5, Heerlen, 6419 PC The Netherlands

**Keywords:** Aging, Arthritis, Rheumatoid, Measures, Outcome, Functional performance, Physical

## Abstract

To investigate whether the association between age and five measures of physical function differs between older adults with rheumatoid arthritis (RA) and population controls. This cross-sectional analysis of the *STudying Ageing in Rheumatoid arthritis* (STAR) study included 207 patients with RA and 214 population controls aged 55–85 years. Physical function was assessed by the self-reported Health Assessment Questionnaire-Disability Index (HAQ-DI) and Cochin hand function scale, and three performance measures including handgrip strength, gait speed and the five-times sit-to-stand test (FTSST) in a subgroup (RA: *n* = 88, controls: *n* = 108). Multivariable regression models assessed associations between age, group (RA/control), and each measure of physical function, adjusting for sociodemographic and clinical factors. Interactions age*group were tested. No meaningful age*group interactions were observed, except for a small effect on the FTSST. Age correlated weakly with measures of physical function in both groups and was, in multivariable analyses, only modestly associated with worse HAQ-DI (β = 0.01/year; 95%CI: 0.01–0.02), higher Cochin hand disability (worsening 2%/year), lower handgrip strength (β = -0.4 kg/year; 95%CI: -0.6 to -0.2), and a slightly slower gait speed (β = -0.01 m/s/year; 95%CI: -0.02 to -0.01). RA was independently associated with worse HAQ-DI (β = 0.12; 95%CI: 0.04–0.21), 40% higher Cochin scores, and lower handgrip strength (β=-3.2 kg; 95%CI:-6.1 to -0.4), but not with gait speed or FTSST. Fatigue and pain were the main covariables attenuating the association of age or group with physical function. Although older adults with RA experience worse self-reported physical function and lower grip strength, this was similar compared to population controls and suggests generic processes of ageing.

**Supplementary Information:**

The online version contains supplementary material available at 10.1007/s00296-026-06204-2.

## Introduction

The global population is ageing rapidly, with the proportion of individuals over 60 years of age expected to almost double from 12% in 2015 to 22% by 2050. During the same period, the number of individuals aged 80 years and above is expected to triple [1]. In the European Union (EU), more than one in five individuals is already 65 years or older [2]. In parallel with population ageing, the number of individuals with rheumatic and musculoskeletal diseases (RMDs), including rheumatoid arthritis (RA), will also increase [3, 4]. 

Ageing is frequently accompanied by a range of negative health-related consequences, including multimorbidity, polypharmacy and geriatric syndromes such as sarcopenia and frailty [3, 5, 6]. Geriatric syndromes often act synergistically with each other and with existing diseases, amplifying their negative impact on physical function and further development of functional disability [7–13]. In turn, this interplay may result in new chronic diseases, loss of independence, and increased mortality risk [14, 15]. 

RA is a chronic autoimmune disease characterized by joint inflammation, pain and joint destruction [16]. Chronic inflammation in RA may promote immunosenescence [17]—the gradual deterioration of the immune system with age—which in turn further contributes to accelerated accrual of additional multimorbidity and geriatric syndromes compared to the general population [18, 19]. 

To better understand the clinical impact of these processes on physical function, objective physical performance measures such as handgrip strength or gait speed are commonly used [20, 21]. Already early in the disease course, patients with RA show a lower grip strength across all age groups compared to the general population (40–80% of the expected strength) [22]. In the general population, grip strength declines with age, whereas in RA it tends to remain stably low [23]. Self-reported physical function, measured by the Health Assessment Questionnaire (HAQ), was also worse at younger ages in patients with RA compared to controls, although scores converged by age 80 [24, 25]. Similarly, a longitudinal study found that individuals with arthritis had poorer physical function – based on self-reported limitations in among others mobility and daily activities – at earlier ages than those without arthritis, but experienced a similar rate of functional decline [26]. Together, these findings suggest that RA may lead to earlier onset of functional disability, but at older ages, disability levels in the general population may approach those observed in patients with RA.

Apart from the studies described above, evidence on the effect of ageing on self-reported and objectively measured upper- and lower-limb function in older patients with RA – particularly in comparison to the general population – remains limited. Specifically, no study has yet evaluated different measures of physical function within the same population. It is important to understand to what extent changes in physical function in older patients purely reflect the effect of RA itself, or are also influenced by age-related changes observed in the general population.

The *STudying Ageing in Rheumatoid arthritis* (STAR) study aims to gain insight into the effect of ageing in patients with RA compared to population controls and includes several instruments to assess self-reported and objectively measured physical function. In this cross-sectional study, STAR data were used to investigate (1) whether the association between age and physical function differs between older patients with RA and population controls, and (2) factors contributing to this potential difference.

## Materials and methods

### Study design and population

The STAR study was designed as a cross-sectional observational study including patients with RA and population-based controls aged 55–85 years. Patients diagnosed with RA by their rheumatologists were recruited from the outpatient clinics of two large hospitals in the south of the Netherlands: Maastricht University Medical Centre + and Zuyderland Medical Centre. Population controls were recruited through patients’ and employees’ friends or family members, snowball sampling and flyers. Presence of an RMD (except for osteoarthritis) was a strict exclusion criterion for population controls. Additional exclusion criteria for all participants were (1) short-term life-threatening disease, (2) a diagnosis of dementia or severe cognitive decline, or (3) inability to understand Dutch or the study information.

**Fig. 1:**
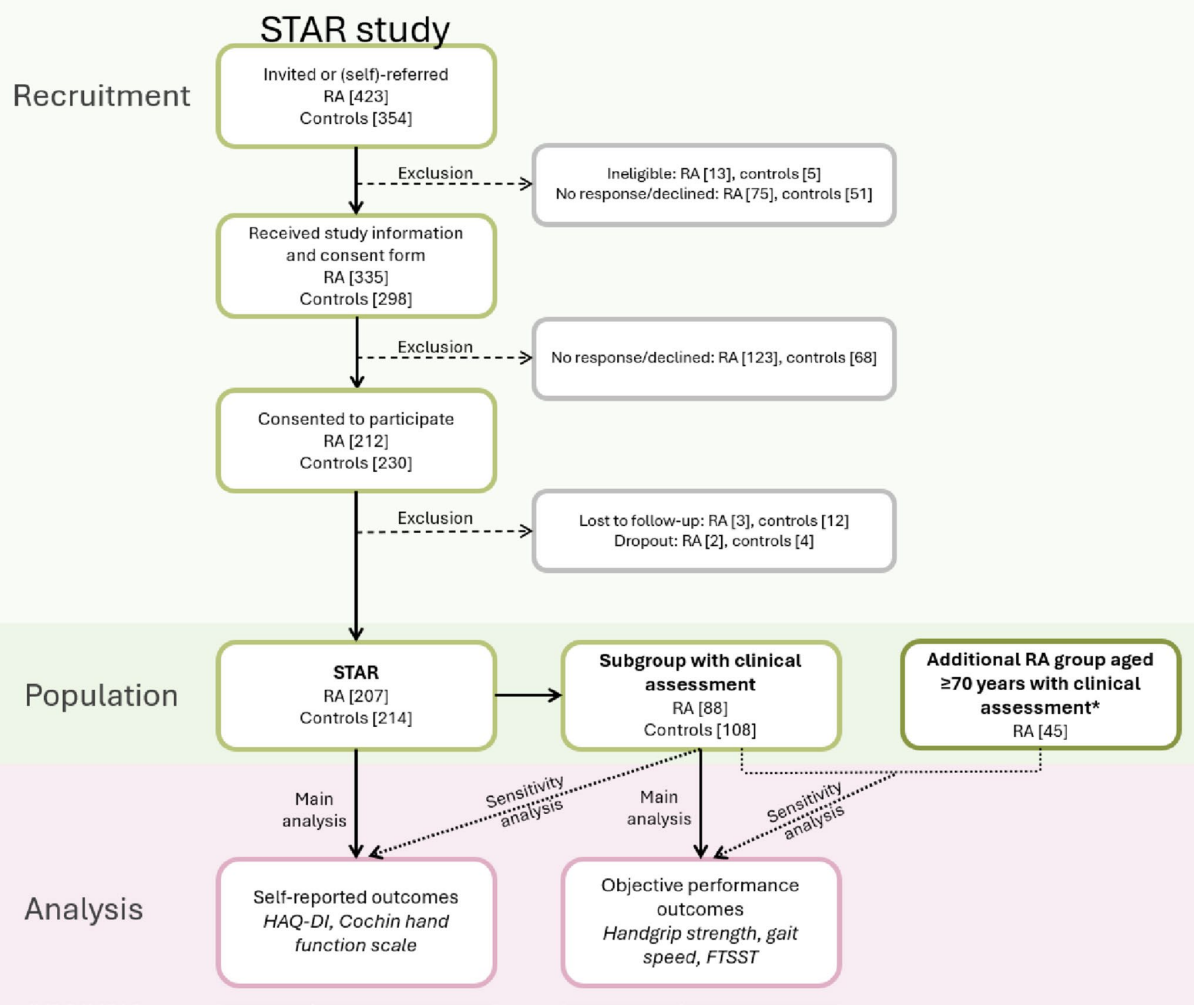
Flowchart of the STAR study. Self-reported outcomes included HAQ-DI and Cochin hand functional disability scale. Objective performance outcomes included HGS, gait speed and FTSST. Covariables included sex, height, education, smoking, BMI, CCI, MFI, VAS pain plus (1) RADAI only for the main STAR population and (2) SJC, TJC, hand joint nodes and CRP only for the subgroup with clinical assessment and additional RA group aged ≥ 70 years. Fatigue (MFI) was not available in the additional RA group aged ≥ 70 years. *Available outcome measures included only HGS and gait speed. *STAR* studying ageing in rheumatoid arthritis; *HAQ-DI* Health Assessment Questionnaire Disability Index; *HGS* handgrip strength; *FTSST* five times sit-to-stand test; *BMI* body mass index; *CCI* Charlson Comorbidity Index; *MFI* multidimensional fatigue inventory; *VAS* Visual Analogue Scale; *RADAI* Rheumatoid Arthritis Disease Activity Index; *SJC* swollen joint count; *TJC* tender joint count; *CRP* C-reactive protein; *RA* rheumatoid arthritis

All participants underwent a telephone interview to collect information on medical history and medication use, followed by self-administered questionnaires completed at home. A subgroup willing to attend the research centre underwent clinical examinations, performance tests, and laboratory assessments. In this subgroup, the aim was to include ten women and ten men in each five-year age interval (55–59, 60–64, 65–69, 70–74, 75–79, 80–85 yrs) to ensure an appropriate representation across age groups. However, recruitment of patients with RA aged ≥ 70 years for the full clinical examination proved challenging. Therefore, additional patients were enrolled under a reduced protocol and included in sensitivity analyses (additional RA group aged ≥ 70 years) (Fig. 1).

The study was approved by the azM/UM medical ethical committee (NL71972.068.019; 1 June 2021), was conducted in accordance with the principles of the Declaration of Helsinki, and all participants provided written informed consent. Recruitment and data collection took place between December 2022 and April 2025.

### Outcome measures

Five outcome measures were considered: two self-reported measures (HAQ-Disability Index (HAQ-DI) and Cochin hand functional disability scale) and three performance-based measures (handgrip strength, gait speed and the five-times sit-to-stand test (FTSST)). Performance measures were assessed only in the subgroup undergoing clinical assessments. In the additional RA group aged ≥ 70 years, only handgrip strength and gait speed were measured. More information on the outcome measures and their clinical relevance is provided in Supplementary Table S1.

#### Health assessment questionnaire-disability index

The self-reported HAQ-DI evaluates difficulty in performing activities of daily living in the past week across eight categories: dressing, arising, eating, walking, hygiene, reach, grip, and common daily activities [27]. Each category consists of 2–3 items, scored from 0 (no difficulty) to 3 (unable to do). The highest item score determines the score for that category. The overall HAQ-DI score is the mean of all category scores, ranging from 0 to 3, with higher scores indicating more disability. If assistive devices or help from another person were reported, a minimum score of 2 was assigned for the relevant item. The total score was calculated when at least six of the eight categories were completed [27]. Impaired physical ability was defined as a score > 1 (mild to severe disability) based on previous research [28]. 

#### Cochin hand functional disability

The self-reported Cochin hand functional disability scale evaluates changes in functional hand disability in RA [29]. It comprises 18 questions on daily activities, scored from 0 (done without difficulty) to 5 (impossible to do). The total score ranges from 0 (no functional hand disability) to 90 (maximal functional hand disability). No validated thresholds for impaired hand function are available.

#### Handgrip strength

Handgrip strength was assessed using a calibrated digital Jamar handheld dynamometer with the handle set to the second position. Participants were seated with forearms in a neutral position, elbows flexed at 90°, and instructed to exert maximum grip strength three times per hand without resting between trials. Hand dominance (left, right or ambidextrous) was recorded. Mean grip strength (kg) was calculated from three measurements for both the dominant and non-dominant hand. In ambidextrous individuals, the strongest hand was considered dominant. Reduced handgrip strength (= lowest 20% of population-based older adults) was defined using gender- and BMI-specific thresholds from the Fried frailty criteria (men: Body Mass Index (BMI) ≤ 24: handgrip strength (HGS) ≤ 29 kg; BMI 24.1–26.0: HGS ≤ 30 kg; BMI 26.1–28.0: HGS ≤ 30 kg; BMI > 28.0: HGS ≤ 32 kg, women: BMI ≤ 23: HGS ≤ 17 kg; BMI 23.1–26.0: HGS ≤ 17.3 kg; BMI 26.1–29.0 HGS ≤ 18 kg; BMI > 29.0: HGS ≤ 21 kg) [30]. 

#### Gait speed

Gait speed was measured using the 4-meter walk test. Participants were instructed to walk at their usual pace from a standing position behind the starting line. Timing started at the first foot movement and ended when both feet crossed the finish line. Walking aids were allowed if needed. Slow walking speed was defined as ≤ 0.8 m/s according to the thresholds proposed by the European Working Group on Sarcopenia in Older People (EWGSOP2) [31]. 

#### Five times sit-to-stand test

The FTSST assesses lower limb muscle strength and balance. Participants rose from a chair with arms folded across their chest and returned to the seated position as quickly as possible for five repetitions. The time of five completed repetitions was recorded by a stopwatch. Low strength based on the FTSST was defined as > 15.0 s according to EWGSOP2 thresholds [31]. 

### Covariables

All participants completed self-administered questionnaires. Sociodemographic and lifestyle characteristics included age (years), sex, educational level (low (no education, primary education, or lower vocational education), middle (intermediate vocational education or higher secondary education), high (higher vocational education or university education)), smoking status (never, former, current), and BMI. General health was rated on a visual analogue scale (VAS; 0 = very bad, 10 = very good), as was pain during the past week (0 = no pain, 10 = unbearable pain). For patients with RA, the Patient Global Assessment (PGA) and, for controls, joint complaints were recorded using a similar VAS (0 = very little, 10 = a lot). Fatigue was assessed with the 20-item Multidimensional Fatigue Inventory (MFI; 1 = yes, that is true, 5 = no, that is not true; total score 20 (no fatigue) to 100 (maximum fatigue)) [32]. The self-reported painful joint score was assessed by the Rheumatoid Arthritis Disease Activity Index (RADAI; 8 joint groups per side, 0 = no pain to 3 = severe pain per group; total score 0 (no joint pain) to 48 (severe pain in all joint groups)) [33]. The MFI and RADAI were not assessed in the additional RA group aged ≥ 70 years.

Detailed information on current medication use and medical history was collected through a structured telephone interview and, when possible, cross-checked with participants’ electronic medical records (EMR). For patients with RA, current and past use of disease-modifying antirheumatic drugs (DMARDs), glucocorticoids and non-steroidal anti-inflammatory drugs (NSAIDs) was recorded. The presence of erosive disease (yes/no) was assessed by reviewing the most recent radiograph of the hands and feet, and if not available, by screening the EMR. Comorbidities were classified using the Charlson Comorbidity Index (CCI; 0 = no comorbidities, 33 = maximum predicted mortality) [34]. For analysis, CCI scores were categorized as 0, 1, or ≥ 2, excluding the presence of RA.

In the subgroup undergoing clinical assessment and in the additional RA group aged ≥ 70 years, joint examination included the Swollen Joint Count-66 (SJC66) and Tender Joint Count-68 (TJC68) to assess 66 joints for swelling and 68 joints for tenderness. In addition, bony swelling of interphalangeal (IP), distal IP (DIP), proximal IP (PIP) and carpometacarpal (CMC) joints of the hands was recorded. A blood sample was collected to measure erythrocyte sedimentation rate (ESR) and C-reactive protein (CRP) level (in the additional RA group aged ≥ 70 years, most recent results were derived from the EMR).

### Statistical analysis

Demographics, clinical characteristics and the five outcomes of interest were described for patients and controls, separately for the main STAR population and both subgroups. Correlations (Pearson or Spearman, as appropriate) between age and the five outcomes of interest were explored separately for patients with RA and population controls, and visualized using scatter plots. Correlation coefficients were interpreted as follows: 0.00 to 0.10 = no correlation, 0.10 to 0.39 = weak, 0.40 to 0.69 = moderate, ≥ 0.70 = strong [35]. 

Manual forward multivariable linear regression models were constructed to assess potential differential associations of age with outcomes between groups (RA vs. controls) and to gain insight into additional determinants and potential confounders. All covariables described in the section ‘Covariables’ were considered (see also Fig. 1). First, the interaction age*group was tested in a model including age, group and sex. Regressions were stratified by group if *p*_interaction_<0.10 and the interaction was considered clinically relevant. Additional covariables were retained in the model if significantly associated with the outcome (*p* < 0.05) or if they acted as confounders by changing the coefficient for age or group by > 10% upon inclusion. Multicollinearity was assessed using variance inflation factors (VIF), with values > 10 indicating high multicollinearity. Missing data were not imputed; analyses included only participants with available outcome and covariable data.

Because of an excess of zero values, Cochin scores were analyzed using a zero-inflated negative binomial (ZINB) regression model. ZINB regression showed a better fit than standard negative binomial regression and zero-inflated Poisson models, as indicated by lower Akaike and Bayesian Information Criterion (AIC and BIC) values.

Three sensitivity analyses were performed. First, to enhance clinical interpretability, multivariable logistic regression analyses were conducted for dichotomous outcomes of impaired ability or performance for each outcome measure based on clinically meaningful thresholds. Second, analyses for handgrip strength and gait speed were repeated, also including the additional RA group aged ≥ 70 years. Third, analyses for HAQ-DI and Cochin were repeated in the subgroup with clinical assessment to understand robustness and compare with questionnaire-only participants (Fig. 1).

Statistical analyses were performed using STATA (StataCorp version 17).

## Results

### Study population

A total of 421 participants were included in STAR of which 207 patients with RA (mean age 67.8 (SD 7.3) yrs; 62% women) and 214 population controls (mean age 68.1 (7.1) yrs; 60% women) (Fig. 1; Table 1). The subgroup with clinical assessment consisted of 196 participants: 88 patients with RA (mean age 67.3 (7.8) yrs; 49% women) and 108 controls (mean age 69.1 (8.1) yrs; 56% women). The additional RA group aged ≥ 70 years included 45 patients with RA (mean age 76.6 (8.9) yrs, 53% women).

Compared to controls, patients with RA had more often a low or middle educational level (58% vs. 42%), were more often smokers (15% vs. 6%) and had more comorbidities (CCI score ≥ 2: 16% vs. 10%). The subgroup with clinical assessment had a more balanced sex distribution compared to the overall study population. When comparing the main study population *without* the clinical assessment subgroup to the subgroup *with* clinical assessment (Supplementary Table S2), patients with RA in the latter subgroup had slightly less multimorbidity (CCI score ≥ 2: 10% vs. 20%). As expected, the additional RA group aged ≥ 70 years had more comorbidities than the main RA population (CCI score ≥ 2: 29% vs. 16%) and a higher proportion of patients receiving glucocorticoids (36% vs. 25%) (Table 1).

### Physical function

In the overall STAR study population, patients with RA showed higher median HAQ-DI (0.6 [IQR 0.3–1.3]) and Cochin hand function scores (6 [1–18]) compared to controls (HAQ-DI: 0.1 [0.0–0.5]; Cochin: 0 [0–3]) (Table 2). In the subgroup with clinical assessments, mean dominant handgrip strength was comparable between women with RA (20.7 (7.4) kg) and controls (22.4 (7.2) kg). In men, those with RA had lower handgrip strength (32.6 (13.4) kg) than controls (38.6 (13.7) kg). Mean gait speed was similar between groups (both 1.1 (0.3) m/s), and the mean time to complete the FTSST was only slightly longer in patients with RA (14.7 (6.6) s) than in controls (13.7 (5.3) s).

### Correlations between age and physical function

**Fig. 2:**
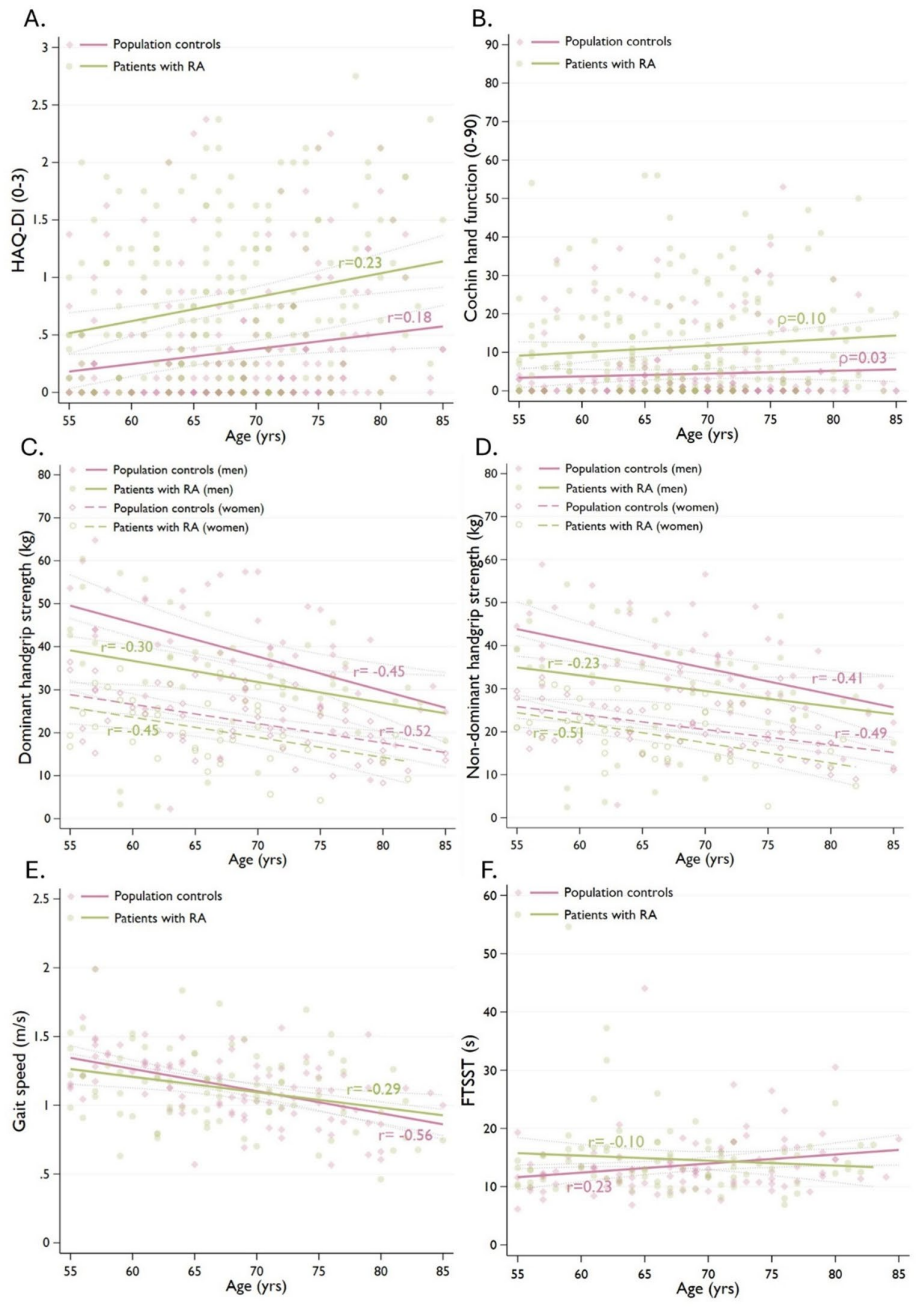
**A**–**F** Five physical function measures by age, stratified for population controls (◇) and patients with RA (○). Main population: **A** HAQ-DI (controls: *n* = 214, *r* = 0.18; RA: *n* = 207, *r* = 0.23), **B** the Cochin hand function scale (controls: *n* = 214, ρ = 0.03; RA: *n* = 205, ρ = 0.10), subgroup with clinical assessment: **C** observed dominant handgrip strength (kg) (controls: n_men_=48, r_men_= -0.46, n_women_=60, r_women_= -0.52; RA: n_men_=45, r_men_= -0.30, n_women_=43, r_women_= -0.45), **D** observed non-dominant handgrip strength (kg) (controls: n_men_=48, r_men_= -0.41, n_women_=60, r_women_= -0.49; RA: n_men_=45, r_men_= -0.23, n_women_=43, r_women_= -0.51), **E** Gait speed (m/s) (controls: *n* = 107, *r*= -0.56; RA: *n* = 86, *r*= -0.29), **F** FTSST (s) (controls: *n* = 94, *r* = 0.23; RA: *n* = 84, *r*= -0.10). *HAQ-DI* Health Assessment Questionnaire Disability Index; *FTSST* five times sit-to-stand test; *RA* rheumatoid arthritis

Correlations with age were weak for HAQ-DI (r_RA_ = 0.23; r_controls_=0.18) and absent for Cochin scores (ρ_RA_ = 0.10; ρ_controls_ = 0.03). Age correlated moderately with dominant and non-dominant handgrip strength, but slightly weaker in patients compared to controls (dominant hand men r_RA_ = -0.30; r_controls_ = -0.46 and women r_RA_=-0.45; r_controls_ = -0.52). For gait speed, correlations with age were weak in RA and moderate in controls (r_RA_= -0.29 r_controls_=-0.56). FTSST showed no correlation with age in RA and only a weak positive correlation in controls (r_RA_ = -0.10; r_controls_ = 0.23) (Fig. 2A–F). Of note, because patients with RA already had worse scores at age 55 for self-reported measures, and the correlation with age was slightly stronger in this group, the regression lines for patients and controls diverged. The opposite pattern was observed for handgrip strength, where the regression lines converged as the association with age was numerically stronger in controls.

### Multivariable associations with physical function

A significant interaction between age and group was present only for the FTSST (*p*_interaction_=0.08). In multivariable analyses, older age had a weak, positive association with a longer FTSST time among controls (although not statistically significant; β_age_ = 0.10, 95%CI: -0.06–0.26), while no association was present in patients with RA (β_age_= -0.02, 95%CI: -0.20–0.17) (Table 3).

Table 3 presents the results of the final models on the relationship between age and RA (vs. controls) with the functional outcomes. Each additional year of age was associated with a 0.01 worsening in HAQ-DI (β_age_ = 0.01, 95%CI: 0.01–0.02), a worsening of 2% in Cochin disability among those with hand disability (Cochin score > 0; count model), worse handgrip strength (β_age_= -0.4, 95%CI: -0.6 to -0.2), and slightly slower gait speed (β_age_= -0.01, 95%CI: -0.02 to -0.01). As noted above, the associations between age and the FTSST in both RA and controls were insignificant.

Compared to controls and while adjusting for confounders, patients with RA reported worse HAQ-DI (β_RA_ = 0.12, 95%CI: 0.04–0.21), were less likely to report no hand disability (Cochin = 0; logit model) (OR_RA_= 0.37, 95% CI: 0.20–0.68), and, among those with hand disability, had 40% higher expected Cochin scores. RA was also associated with lower handgrip strength (β_RA_= -3.2, 95%CI: -6.1 to -0.4), but not with gait speed or FTSST.

Additional associations were quite inconsistent and included *female sex* for worse HAQ-DI and handgrip strength, *middle/higher education* for worse Cochin hand function, *higher comorbidity score* for better Cochin hand function and worse FTSST (controls only), *higher RADAI joint score* or *TJC* for worse HAQ-DI, Cochin hand function, handgrip strength and FTSST, *higher CRP* for worse handgrip strength, *higher MFI score* for worse HAQ-DI, Cochin hand function, gait speed and FTSST (controls only), and *higher VAS pain* for worse Cochin hand function.

### Sensitivity analyses

When dichotomizing the function outcomes according to pre-defined thresholds, an age*group interaction was now found for Cochin hand function (*p*_interaction_=0.09) instead of FTSST. Age increased the odds of any hand disability (Cochin ≥ 1) in RA (OR_age_=1.07, 95% CI: 1.01–1.13), but not in controls (OR_age_= 1.00, 95% CI: 0.95–1.05) (Supplementary Table S3). Comparable to linear regression, age increased the odds of mild to severe HAQ-DI, diminished handgrip strength (Fried criteria), and slow gait speed. Moreover, RA remained associated with HAQ-DI > 1 and diminished handgrip strength.

When including the additional RA group aged ≥ 70 years, women with RA showed a slightly steeper age-related decline (Supplementary Figure S1A). The correlation between age and gait speed became slightly stronger (from *r*=-0.29 to *r*=-0.36) (Supplementary Figure S1B). The final models were largely consistent with the main analyses (Supplementary Table S4), although the effect of group (RA vs. control) became stronger.

When limiting the regression models for HAQ-DI and Cochin to the subgroup with clinical assessments, results were largely consistent with the main analyses (including all participants), suggesting robustness of the findings (Supplementary Table S5).

## Discussion

This cross-sectional analysis of the STAR study found that the association between age and both self-reported and performance-based physical function did not differ between patients with RA and population controls. The strongest correlation with age was seen for handgrip strength, but overall, correlations were weak. However, age remained significantly associated with all outcomes in multivariable analyses, except for FTSST. The additional negative effect of RA on overall physical function (HAQ-DI), hand disability (Cochin) and handgrip strength illustrates that older RA patients have more limitations in physical function. Still, the age-related differences in these outcomes appear comparable to that observed in the general population, possibly reflecting effective RA management, as many patients were treated with cs- or bDMARDs. Interestingly, gait speed and FTSST – reflecting lower-limb performance – were largely similar between RA and controls, suggesting that RA has little additional impact on these measures beyond “normal” ageing.

Few studies have compared different measures of physical function between patients with RA and population controls across age. The absence of a statistically significant differential association of age with the HAQ-DI in our study contrasts two previous reports that observed convergence of HAQ scores and disability around the age of 80 [24, 25]. In our patients, HAQ-DI seemed to worsen slightly more than in controls, although this divergence was not statistically significant. As for handgrip strength, a systematic review reported age-related declines in the general population, particularly after age 50, whereas in patients with RA, handgrip strength was consistently lower but not associated with age [23]. This review concerned a younger RA population (31–65 yrs). We included an older age range (55–85 yrs), and observed age-related declines in handgrip strength in both groups, with values appearing to converge at older ages in men. Enriching the analyses with additional RA patients aged ≥ 70 years did not change these findings. A reduced gait speed in RA was reported in previous studies [36], but became only significant in our study when including the additional group of older RA patients. This is likely a result of insufficient adjustment for confounding, as fatigue – an important confounder for the effect of RA on outcomes – was not measured in this additional group. For the FTSST, one previous study found worse performance in RA compared to controls using a comparable sit-to-stand instrument [37]. In our study, FTSST performance was similar across age and appeared to worsen slightly in controls, although this association was not statistically significant. The absence of an age-related association for the FTSST in patients with RA raises questions about its validity as a measure of age-related functional differences in older adults with RA. Moreover, it is possible that gait speed and the FTSST capture broader aspects of physical function, including balance, coordination, and cardiovascular fitness, which may outweigh the contribution of joint-related impairment.

To our knowledge, this is the first study to show that the association between age and physical function is weak and comparable between patients with RA and controls. Based on the concepts of inflammaging and immunosenescence, which suggest accelerated ageing processes in RA, we expected patients with RA to show a steeper age-related decline in physical function than the general population. However, our results do not support this hypothesis, as no clinically relevant differences were found between the groups. This may reflect advances in RA management, particularly the use of DMARDs, which help preserve physical function. However, despite these therapeutic improvements, patients with RA often still experience symptoms such as fatigue or pain, which may contribute to functional limitations independent of inflammatory activity. Fatigue and joint pain were the main covariables attenuating the association between age or RA and self-reported and performance-based physical function, although the effect of RA itself remained significant even after adjusting for disease-related factors. Since these symptoms are also common in the general population due to age-related conditions as osteoarthritis or sarcopenia, they may partly explain the similar age-related decline observed in both groups.

Several limitations should be acknowledged. First, the subgroup undergoing objective outcome measurements may not fully represent the general RA population, as healthier patients were more likely to visit the hospital for these measurements, potentially introducing selection bias. The assessed subgroup had slightly less multimorbidity compared to those who only completed questionnaires (CCI score ≥ 2: 10% vs. 20%), which may have led to underestimation of physical limitations in performance-based measures. However, sensitivity analysis suggests negligible effects. Furthermore, sensitivity analyses including additional patients aged ≥ 70 years who declined full participation did not substantially alter conclusions for handgrip strength and gait speed. Conversely, selection bias among controls may have favoured less healthy individuals, which could lead to underestimation of differences between RA patients and controls. Moreover, the recruitment of population controls with help of snowball sampling and flyers may limit the generalisability of findings to the broader general population. However, we prioritised internal above external validity. Second, the small number of participants aged 80–85 limits generalizability for the oldest old. Third, we were unable to include disease duration or age at RA diagnosis in the models, as these variables are not applicable to controls. Fourth, the use of cross-sectional data precludes our ability to demonstrate causal relationships between age and physical function.

Strengths of this study include detailed data collection in older patients with RA and population controls in a real-world setting. The inclusive design, which did not exclude participants with multiple or severe comorbidities, further enhances the external validity and applicability of our findings to clinical practice. Moreover, the concomitant inclusion of self-reported and performance-based measures was a strength, as they seem to capture distinct yet complementary aspects of physical function [38]. 

**Table 1:** Characteristics of the main STAR study population, subgroup with clinical assessment, and additional RA group aged ≥ 70 years

	Main study population (*n* = 421)		Subgroup with clinical assessment (*n* = 196)		Additional RA group aged ≥ 70 years (*n* = 45)
	RA *n* = 207	Control *n* = 214	RA *n* = 88	Control *n* = 108	RA *n* = 45
Age (yrs), mean (SD)	67.8 (7)	68.1 (7)	67.3 (8)	69.1 (8)	76.6 (9)
Sex, n women (%)	128 (62)	128 (60)	43 (49)	60 (56)	24 (53)
Educational level, n (%)^a^					
Low	46 (22)	20 (9)	18 (21)	14 (13)	11 (24)
Middle	73 (35)	69 (32)	35 (40)	37 (34)	20 (44)
High	86 (42)	123 (58)	34 (39)	56 (52)	11 (24)
Smoking status, n (%)					
Never	77 (37)	102 (48)	31 (35)	54 (50)	13 (29)
Former	100 (48)	100 (47)	47 (53)	48 (44)	28 (62)
Current	30 (15)	12 (6)	9 (10)	5 (5)	4 (9)
BMI (kg/m^2^), mean (SD)	26.5 (4)	26.5 (6)	27.0 (5)	27.7 (7)	26.6 (4)
Disease duration (yrs), median (IQR)	7 (3–16)	-	10 (3–20)	-	18 (10–31)
Erosive disease, n *yes* (%)^b^	57 (28)	-	35 (40)	-	25 (56)
Current RA treatment, n (%)					
csDMARDs	156 (75)	-	68 (77)	-	28 (62)
bDMARDs	74 (36)	-	34 (39)	-	18 (40)
tsDMARDs	4 (2)	-	1 (1)	-	1 (2)
Glucocorticoids	51 (25)	-	19 (22)	-	16 (36)
NSAIDs	43 (21)	-	22 (25)	-	9 (20)
RADAI score (0–48), median (IQR)	10 (5–18)	3 (1–8)	11 (5–16)	4 (1–10)	-
Self-rated general health (0–10), mean (SD)	6.5 (1)	7.4 (1)	6.4 (1)	7.2 (1)	5.9 (2)
PGA (0–10), mean (SD)	4.3 (2)	3.0 (3)	4.0 (2)	3.4 (3)	3.7 (3)
VAS pain (0–10), mean (SD)	4.1 (2)	2.7 (3)	4.0 (2)	3.3 (3)	4.4 (3)
MFI (20–100), median (IQR)^c^	52 (41–63)	37 (28–54)	52 (44–63)	37 (29–56)	-
CCI (0–32), n *score* (%)^d^					
0	117 (57)	150 (70)	54 (61)	75 (69)	20 (44)
1	57 (28)	43 (20)	25 (28)	20 (19)	12 (27)
≥ 2	33 (16)	21 (10)	9 (10)	13 (12)	13 (29)
Height (cm), mean (SD)	-	-	172 (11)	171 (9)	168 (10)
CRP (mg/mL), median (IQR)^e^	-	-	3 (1–8)	1 (1–3)	3 (1–11)
SJC (0–66), median (IQR)^f^	-	-	0 (0–1)	0 (0–0)	0 (0–1)
TJC (0–68), median (IQR)^f^	-	-	1 (0–4)	0 (0–1)	2 (0–6)
Joint nodes hands, median (IQR)^g^	-	-	3 (1–8)	6 (2–10)	2 (1–6)

*RA* rheumatoid arthritis; *BMI* body mass index; *csDMARDs* conventional synthetic disease-modifying anti-rheumatic drugs; *bDMARDs* biologic disease-modifying anti-rheumatic drugs; *tsDMARDs* targeted synthetic disease-modifying anti-rheumatic drugs; *NSAID* non-steroidal anti-inflammatory drugs; *RADAI* rheumatoid arthritis disease activity index; *PGA* Patient Global Assessment; *HAQ-DI* Health Assessment Questionnaire Disability Index; *MFI* multidimensional fatigue inventory; *CCI* charlson comorbidity index; *CRP* C-reactive protein; *SJC* swollen joint count; *TJC* tender joint count

^a^Unknown educational level: *n* = 2 (RA) and *n* = 2 (controls) in main study population, *n* = 1 (RA) and *n* = 1 (controls) in subgroup, and *n* = 1 (RA) in additional RA group aged ≥ 70 yrs

^b^Unknown presence of erosive disease: *n* = 29 in main study population, *n* = 4 in subgroup and *n* = 4 in additional RA group aged ≥ 70 yrs

^c^Unknown MFI: *n* = 1 (RA) and *n* = 13 (controls) in subgroup

^d^Rheumatic disease was not included in the CCI

^e^Unknown CRP: *n* = 3 (RA) and *n* = 17 (controls) in subgroup and *n* = 3 (RA) in additional RA group aged ≥ 70 yrs

^f^Unknown SJC and TJC: *n* = 15 (controls)

^g^Unknown joint nodes in the hands: *n* = 13 (controls)

**Table 2:** Outcome measures of physical function in the main STAR study population, subgroup with clinical assessment, and additional RA group aged ≥ 70 years

	Main study population (*n* = 421)			Subgroup with clinical assessment (*n* = 196)			Additional RA group aged ≥ 70 years (*n* = 45)
	RA *n* = 207	Controls *n* = 214	*p*-value	RA *n* = 88	Controls *n* = 108	*p*-value	RA *n* = 45
Sex, n women (%)	128 (62)	128 (60)	0.67	43 (49)	60 (56)	0.38	24 (53)
HAQ-DI (0–3), median (IQR)^a^	0.6 (0.3–1.3)	0.1 (0.0–0.5)	< 0.01	0.6 (0.1–1.3)	0.1 (0.0–0.6)	< 0.01	-
HAQ-DI (0–3), n score >1 (%)	68 (33)	24 (11)	< 0.01	28 (32)	12 (13)	< 0.01	-
Cochin hand function scale (0–90), median (IQR)^b^	6 (1–18)	0 (0–3)	< 0.01	5 (0–18)	0 (0–3)	< 0.01	-
Cochin hand function scale (0–90), n score ≥ 1 (%)	154 (75)	87 (41)	< 0.01	63 (72)	37 (39)	< 0.01	-
Handgrip strength (kg), mean (SD)^c^							
Women: dominant hand	-	-	-	20.7 (7)	22.4 (7)	0.24	12.8 (8)
Women: non-dominant hand	-	-	-	19.2 (7)	20.8 (6)	0.22	12.4 (8)
Men: dominant hand	-	-	-	32.6 (13)	38.6 (14)	0.04	28.8 (12)
Men: non-dominant hand	-	-	-	30.1 (13)	35.5 (12)	0.04	27.3 (12)
Handgrip strength frailty criterion, n (%)^d^							
Women: dominant hand	-	-	-	17 (40)	18 (30)	0.31	20 (83)
Women: non-dominant hand	-	-	-	22 (51)	19 (31)	0.05	20 (83)
Men: dominant hand	-	-	-	18 (40)	13 (27)	0.19	9 (53)
Men: non-dominant hand	-	-	-	22 (49)	13 (27)	0.03	9 (53)
Gait speed (m/s), mean (SD)^e^	-	-	-	1.1 (0.3)	1.1 (0.3)	0.59	1.0 (0.2)
Gait speed (m/s), *n* < 0.8 m/s (%)	-	-	-	14 (16)	14 (13)	0.53	14 (31)
Five times sit-to-stand test (s), mean (SD)	-	-	-	14.7 (6.6)	13.7 (5.3)	0.17	-
Five times sit-to-stand test (s), *n* > 15.0 s (%) ^f^	-	-	-	31 (37)	25 (27)	0.14	-

*RA* rheumatoid arthritis; *HAQ-DI* Health Assessment Questionnaire Disability Index; *HGS* handgrip strength; *FTSST* five times sit-to-stand test

^a^Unknown HAQ-DI: *n* = 1 (RA) and *n* = 13 (controls) in subgroup, as not all controls with physical measurements completed all questionnaires

^b^Unknown Cochin hand function scale: *n* = 2 (RA) in main study population, *n* = 1 (RA) and *n* = 13 (controls) in subgroup, as not all controls with physical measurements completed all questionnaires

^c^Unknown handgrip strength: *n* = 4 (RA) in additional RA group aged ≥ 70 yrs

^d^Based on the threshold for dominant hand grip strength of the Fried frailty criteria, stratified for gender and BMI quartiles: *Men* BMI ≤ 24 HGS ≤ 29 kg, BMI 24.1–26.0 HGS ≤ 30 kg, BMI 26.1–28.0 HGS ≤ 30 kg, BMI > 28.0 HGS ≤ 32 kg, *Women* BMI ≤ 23 HGS ≤ 17 kg, BMI 23.1–26.0 HGS ≤ 17.3 kg, BMI 26.1–29.0 HGS ≤ 18 kg, BMI > 29.0 HGS ≤ 21 kg

^e^Unknown gait speed: *n* = 3 (RA) in subgroup, *n* = 12 (RA) in additional RA group aged ≥ 70 yrs; ^f^Unknown FTSST: *n* = 4 (RA) and *n* = 14 (controls)

**Table 3:** Associations between age, group (RA vs. controls) and measures of physical function, adjusted for sociodemographic and clinical factors, stratified by group when an interaction age*group was present – multivariable linear and ZINB regression

	HAQ-DI	Cochin hand function scale		Dominant handgrip strength (kg)	Gait speed (m/s)	Five times sit-to-stand test (s)	
	Total (*n* = 420)	Total (*n* = 420)		Total (*n* = 175)	Total (*n* = 173)	RA (*n* = 80)	Controls (*n* = 90)
	β (95% CI)	Logit model^a^ OR (95% CI)	Count model^b^ IRR (95% CI)	β (95% CI)	β (95% CI)	β (95% CI)	β (95% CI)
Age, years	**0.01 (0.01–0.02)**	-	**1.02 (1.002–1.038)**	**-0.4 (-0.6 to -0.2)**	**-0.01 (-0.02 to -0.01)**	-0.02 (-0.20–0.17)	0.10 (-0.06–0.26)
Group, patient with RA	**0.12 (0.04–0.21)**	**0.37 (0.20–0.68)**	**1.40 (1.08–1.82)**	**-3.2 (-6.1 to -0.4)**	-0.001 (-0.08–0.08)	NA	NA
Sex, women	**0.22 (0.13–0.30)**	0.55 (0.30–1.02)	1.27 (0.97–1.66)	**-12.1 (-15.7 to -8.5)**	-0.08 (-0.18–0.02)	-0.03 (-2.81–2.75)	0.92 (-2.00–3.84)
Height, cm	NA	NA	NA	**0.24 (0.05 to 0.42)** ^**c,e**^	0.003 (-0.003–0.008)^d^	-	0.02 (-0.15–0.19)
Educational level							
Low	-	Reference	-	-	Reference	Reference	-
Middle	-	**0.29 (0.12–0.71)** ^**d,e**^	-	-	0.08 (-0.03–0.20)^d, e^	-1.3 (-5.3–2.8)^c^	^-^
High	-	**0.27 (0.11–0.63)** ^**d,e**^	-	-	0.03 (-0.08–0.14)^d^	-1.2 (-5.5–3.0)^c^	^-^
Smoking status							
No	-	-	-	-	Reference	-	Reference
Former	-	-	-	-	0.05 (-0.03–0.12)^d^	-	-1.00 (-3.01–1.01)^c^
Current	-	-	-	-	0.07 (-0.08–0.23)^d^	-	-0.002 (-5.91–5.90)^c^
BMI, kg/m^2^	0.01 (-0.001–0.01)^e^	-	-	-	-0.003 (-0.009–0.003)^d^	-	-
CCI (0–32)							
0	Reference	Reference	Reference	Reference	Reference	-	Reference
1	0.01 (-0.09–0.11)^c^	0.63 (0.30–1.33)^e^	0.81 (0.60–1.09)^c^	-0.9 (-4.3–2.4)^c^	-0.03 (-0.12–0.07)^c, d^	-	-0.20 (-3.02–2.62)^c^
≥ 2	0.13 (-0.01–0.26)^c, e^	0.50 (0.17–1.48)^e^	**0.67 (0.46–0.96)** ^**c**^	-2.1 (-6.9–2.8)^c, e^	-0.12 (-0.25–0.02)^c, d,e^	-	**3.78 (0.23–7.32)** ^**c,e**^
RADAI score (0–48)	**0.03 (0.02–0.03)** ^**d,e**^	**0.84 (0.79–0.91)** ^**d,e**^	**1.03 (1.02–1.05)** ^**c,d,e**^	NA	NA	NA	NA
TJC (0–68)	NA	NA	NA	**-0.5 (-0.7 to -0.3)** ^**c,e**^	-	**0.5 (0.3–0.7)** ^**c,e**^	**0.15 (0.02–0.27)** ^**c,e**^
SJC (0–66)	NA	NA	NA	-	-	-0.2 (-0.9–0.5)^c^	-
Joint nodes hands	NA	NA	NA	-	0.004 (-0.004–0.012)^d^	-0.1 (-0.5–0.2)^c^	-
CRP (mg/mL)	NA	NA	NA	**-0.2 (-0.3 to -0.03)** ^**d,e**^	-	0.07 (-0.03–0.17^c^	0.08 (-0.18–0.34)^c^
MFI (20–100)	**0.01 (0.01–0.01)** ^**d,e**^	0.98 (0.96–1.00)^d^	**1.01 (1.002–1.020)** ^**e**^	-0.07 (-0.16–0.02)^d^	**-0.004 (-0.006 to -0.001)** ^**d,e**^	0.06 (-0.05–0.16)^c^	**0.12 (0.06–0.18)** ^**c,e**^
VAS pain (0–10)	-	-	**1.09 (1.02–1.16)** ^**e**^	-	-	-0.2 (-0.8–0.5)^c^	-

*ZINB* zero-inflated negative binomial; *HAQ-DI* Health Assessment Questionnaire Disability Index; *OR* odds ratio; *IRR* incidence rate ratio; *RA* rheumatoid arthritis; *NA* not available/applicable; *BMI* body mass index; CCI Charlson Comorbidity Index; *RADAI* Rheumatoid Arthritis Disease Activity Index; *TJC* tender joint count; *SJC* swollen joint count; *CRP* C-reactive protein; MFI multidimensional fatigue inventory; *VAS* Visual Analogue Scale; Statistically significant values are indicated in bold

^a^Represents how independent variables are related to the likelihood of reporting no hand disability (Cochin score = 0). OR < 1 means someone is less likely to report no disability

^b^Represents how independent variables affect the Cochin score, among those who report any hand disability (score > 0). Incidence Rate Ratio (IRR) indicates the change (%) in score

^c^Confounding variable in the association between age and the outcome measure (∆β_age_ ≥ 10%)

^d^Confounding variable in the association between group and the outcome measure (∆β_group_ ≥ 10%)

^e^Associated with the outcome measure (*p* < 0.05)

In conclusion, although RA is associated with a persistent functional disadvantage in older adult patients, the association between age and the modest decline in functional ability did not differ compared to the general population. Our findings suggest that the age-related differences in physical function may reflect generic age-related rather than RA disease-specific processes, provided patients are treated according to the standard-of-care. Therefore, in older adults in general, the window of opportunity to improve and preserve function may lie in addressing geriatric syndromes such as sarcopenia and frailty. However, for younger patients, early and effective disease control with appropriate therapy may help to preserve long-term function. Future longitudinal research, including larger cohorts of older patients, is needed to confirm and extend these findings.

### Congress abstract publication

Truijen S, Boonen A, Ramiro S, van Onna M. Physical Function Across Age in Patients With Rheumatoid Arthritis and Population Controls: A Cross-Sectional Study of Four Performance Measures [abstract]. *Arthritis Rheumatol.* 2025; 77 (suppl 9). https://acrabstracts.org/abstract/physical-function-across-age-in-patients-with-rheumatoid-arthritis-and-population-controls-a-cross-sectional-study-of-four-performance-measures/.

## Supplementary Information

Below is the link to the electronic supplementary material.

Supplementary Material 1

## Declarations

### Disclaimer

No part of this report is copied or published elsewhere in whole or in part.
