# Supplementary material for: Physical function in older people with rheumatoid arthritis and population controls: a cross-sectional study of self-reported and performance-based measures
Source: Rheumatol Int. 2026 Jun 17;46(7):155. doi: 10.1007/s00296-026-06204-2 (PMC13275541; doi:10.1007/s00296-026-06204-2)
Supplement: Supplementary file 1 — Supplementary Material 1 [file 296_2026_6204_MOESM1_ESM.docx]

**Physical function in older people with rheumatoid arthritis and population controls: A cross-sectional study of self-reported and performance-based measures – Supplementary Material**

Saskia P.M. Truijen, MSc ^a,b^, Annelies Boonen, MD PhD ^a,b^, Sofia Ramiro, MD PhD ^c,d^, Marloes van Onna, MD PhD ^a,b^

^a^ Department of Rheumatology, Maastricht University Medical Center+, P. Debyelaan 25, 6229 HX, Maastricht, The Netherlands
^b^ Care and Public Health Research Institute (CAPHRI), Minderbroedersberg 4-6, 6211 LK, Maastricht University, Maastricht, The Netherlands

^c^ Department of Rheumatology, Leiden University Medical Center, Albinusdreef 2, 2333 ZG, Leiden, The Netherlands
^d^ Department of Rheumatology, Zuyderland Medical Center, Henri Dunantstraat 5, 6419 PC, Heerlen, The Netherlands

Corresponding author: S.P.M. Truijen, Department of Rheumatology, Maastricht University Medical Centre+, P. Debyelaan 25, 6229 HX, Maastricht, The Netherlands, [saskia.truijen@maastrichtuniversity.nl](mailto:saskia.truijen@maastrichtuniversity.nl), +31(0)43-3877024 (secretary), ORCID 0000-0003-1252-5026

| Supplementary Table S1. Clinical relevance of the five outcome measures | | |
| --- | --- | --- |
| **Outcome measure** | **Measurement** | **Interpretation** |
| HAQ-DI | Self-reported physical ability | Higher scores indicate poorer functional ability and a higher likelihood of adverse long-term outcomes (e.g. higher mortality risk [1], higher risk of work disability [2], loss of independence, increased healthcare utilization). |
| Cochin hand disability Scale | Self-reported physical ability | Higher scores indicate poorer hand functional ability in daily activities and changes in patient-perceived handicap.[3] Strongly correlated with HAQ [4] and sensitive to hand surgery outcomes in RA.[5] |
| Handgrip strength | Objective physical performance | One of the major factors for diagnosis of sarcopenia.[6] Lower values indicate reduced muscle strength, and have a predictive validity for decline in cognition, mobility, functional status, falls and mortality.[7] |
| Gait speed | Objective physical performance | Predictor of disability, cognitive decline, falls, hospitalization, nursing home admission and mortality.[8]  Average gait speed in community-dwelling older adults range from 0.60-1.45 m/s with a desired gait speed often cited as 1.2 m/s, the speed required to cross most intersections.[9] |
| Five Times Sit-to-Stand Test | Objective physical performance | Predictor of falls, disability, hospitalization and mortality.[10] Longer times indicate poorer leg strength [11,12] and balance dysfunction [13]. |
| **HAQ-DI** Health Assessment Questionnaire Disability Index; **FTSST** Five-Times Sit-to-Stand Test | | |

| Supplementary Table S2. Comparison of characteristics between the main study population excluding the subgroup with clinical assessment to the subgroup with clinical assessment | | | | | | |
| --- | --- | --- | --- | --- | --- | --- |
|  | **Main study population without subgroup with clinical assessment** | **Subgroup with clinical assessment** |  | **Main study population without subgroup with clinical assessment** | **Subgroup with clinical assessment** |  |
|  | RA  n=120 | RA  n=88 | *p*-value | Control  n=119 | Control  n=108 | *p*-value |
| Age (yrs), mean (SD) | 68.1 (6.9) | 67.3 (7.8) | 0.40 | 68.2 (6.7) | 69.1 (8.1) | 0.35 |
| Sex, n *women* (%) | 85 (71) | 43 (49) | <0.01 | 75 (63) | 60 (56) | 0.25 |
| Educational level, n (%) ^1^ |  |  | 0.68 |  |  | 0.59 |
| Low | 28 (23) | 18 (21) |  | 9 (8) | 14 (13) |  |
| Middle | 38 (32) | 35 (40) |  | 41 (35) | 37 (34) |  |
| High | 52 (43) | 34 (39) |  | 68 (57) | 56 (52) |  |
| Smoking status, n (%) |  |  | 0.24 |  |  | 0.58 |
| Never | 46 (38) | 31 (35) |  | 53 (45) | 54 (50) |  |
| Former | 53 (44) | 47 (53) |  | 59 (50) | 48 (44) |  |
| Current | 21 (18) | 9 (10) |  | 7 (6) | 5 (5) |  |
| BMI (kg/m^2^), mean (SD) | 26.0 (3.9) | 27.0 (4.7) | 0.10 | 25.5 (4.0) | 27.7 (7.2) | <0.01 |
| Disease duration (yrs), median (IQR) | 7.3 (2.9–13.2) | 9.5 (3.3–19.8) | 0.11 | - | - | - |
| Erosive disease, n *yes* (%) ^2^ | 23 (19) | 35 (40) | 0.01 | - | - | - |
| Current RA treatment, n (%) |  |  |  |  |  |  |
| csDMARDs | 89 (74) | 68 (77) | 0.61 | - | - | - |
| bDMARDs | 41 (34) | 34 (39) | 0.51 | - | - | - |
| tsDMARDs | 3 (3) | 1 (1) | 0.48 | - | - | - |
| Glucocorticoids | 32 (27) | 19 (22) | 0.40 | - | - | - |
| NSAIDs | 21 (18) | 22 (25) | 0.19 | - | - | - |
| RADAI score (0–48), median (IQR) | 10 (4–20) | 11 (5–16) | 0.93 | 3 (1–6) | 4 (1–10) | 0.37 |
| Self-rated general health (0–10), mean (SD) | 6.5 (1.3) | 6.4 (1.2) | 0.95 | 7.5 (1.2) | 7.2 (1.3) | 0.15 |
| PGA (0–10), mean (SD) | 4.5 (2.5) | 4.0 (2.2) | 0.21 | 2.6 (2.5) | 3.4 (3.0) | 0.02 |
| VAS pain (0–10), mean (SD) | 4.2 (2.4) | 4.0 (2.3) | 0.54 | 2.3 (2.3) | 3.3 (2.8) | <0.01 |
| MFI (20–100) ^3^ , median (IQR) | 54 (38–64) | 52 (44–63) | 0.70 | 35 (27–50) | 37 (29–56) | 0.38 |
| CCI (0–32), n *score* (%) ^4^ |  |  | 0.16 |  |  | 0.62 |
| 0 | 64 (53) | 54 (61) |  | 79 (66) | 75 (69) |  |
| 1 | 32 (27) | 25 (28) |  | 28 (24) | 20 (19) |  |
| ≥2 | 24 (20) | 9 (10) |  | 12 (10) | 13 (12) |  |
| Cochin hand function scale (0–90), median (IQR) ^5^ | 7 (1–18) | 5 (0–18) | 0.46 | 0 (0–3) | 0 (0–3) | 0.77 |
| Cochin hand function scale (0–90), n *score* *≥1* (%) | 91 (76) | 63 (72) | 0.44 | 50 (42) | 37 (39) | 0.65 |
| HAQ-DI (0–3), median (IQR) ^6^ | 0.6 (0.3–1.3) | 0.6 (0.1–1.3) | 0.79 | 0.1 (0–0.4) | 0.1 (0.0–0.6) | 0.28 |
| HAQ-DI (0–3), n *score* >*1* (%) | 40 (33) | 28 (32) | 0.86 | 12 (10) | 12 (13) | 0.56 |
| ^1^ Unknown educational level: n=2 (RA) and n=1 (controls) in main study population, n=1 (RA) and n=1 (controls) in subgroup; ^2^ Unknown presence of erosive disease: n=25 in main study population, n=4 in subgroup; ^3^ Unknown MFI: n=1 (RA) and n=13 (controls) in subgroup; ^4^ Rheumatic disease was not included in the CCI. ^5^ Unknown Cochin hand function scale: n=2 (RA) in main study population, n=1 (RA) and n=13 (controls) in subgroup, as not all controls with physical measurements completed all questionnaires; ^6^ Unknown HAQ-DI: n=1 (RA) and n=13 (controls) in subgroup, as not all controls with physical measurements completed all questionnaires.  **RA** Rheumatoid Arthritis; **BMI** Body Mass Index; **csDMARDs** Conventional Synthetic Disease-Modifying Anti-Rheumatic Drugs; **bDMARDs** Biologic Disease-Modifying Anti-Rheumatic Drugs; **tsDMARDs** Targeted Synthetic Disease-Modifying Anti-Rheumatic Drugs; **NSAID** Non-Steroidal Anti-Inflammatory Drugs; **RADAI** Rheumatoid Arthritis Disease Activity Index; **PGA** Patient Global Assessment; **MFI** Multidimensional Fatigue Inventory; **CCI** Charlson Comorbidity Index; **HAQ-DI** Health Assessment Questionnaire Disability Index | | | | | | |

| Supplementary Table S3. Associations between age, group (RA vs. controls) and clinical thresholds of diminished physical function, adjusted for sociodemographic and clinical factors, stratified by group when an interaction age*group was present – multivariable logistic regression | | | | | | |
| --- | --- | --- | --- | --- | --- | --- |
|  | **HAQ-DI >1 vs. ≤1** OR (95% CI) n=419 | **Cochin hand function scale 0 vs. ≥1** OR (95% CI) n=419 | | **Dominant handgrip  strength (kg) Fulfil Fried criteria vs. not fulfil Fried criteria** OR (95% CI) n=174 | **Gait speed (m/s) <0.8 vs. ≥0.8 m/s** OR (95% CI) n=174 | **Five times  sit-to-stand test (s) >15 vs. ≤15 s** OR (95% CI) n=174 |
|  |  | RA  (n=205) | Controls  (n=214) |  |  |  |
| Age, *years* | **1.07 (1.02–1.11)** | **1.07 (1.01–1.13)** | 1.00 (0.95–1.05) | **1.14 (1.07–1.20)** | **1.12 (1.04–1.21)** | 1.04 (0.98–1.09) |
| Group, *patient with RA* | **2.31 (1.17–4.57)** | NA | NA | **2.58 (1.19–5.63)** | 2.68 (0.88–8.17) | 1.19 (0.56–2.53) |
| Sex, *women* | **3.98 (1.87–8.46)** | **2.52 (1.12–5.66)** | 1.63 (0.81–3.27) | 1.22 (0.56–2.67) | 0.99 (0.34–2.81) | 1.23 (0.58–2.60) |
| Height, *cm* | NA | NA | NA | - | - | - |
| Educational level |  |  |  |  |  |  |
| Low | - | - | - | *reference* | - | *reference* |
| Middle | - | - | - | 0.76 (0.26–2.16) | - | **0.27 (0.09–0.83) ^3^** |
| High | - | - | - | **0.28 (0.10–0.82) ^3^** | - | 0.58 (0.21–1.58) ^3^ |
| Smoking status |  |  |  |  |  |  |
| No | - | *reference* | - | - | *reference* | - |
| Former | - | 0.54 (0.22–1.31) | - | - | **0.30 (0.09–0.94) ^2,3^** | - |
| Current | - | **0.20 (0.06–0.67) ^3^** | - | - | 0.36 (0.03–4.39) ^2^ | - |
| BMI, *kg/m^2^* | 1.02 (0.97–1.08)^3^ | - | - | - | - | - |
| CCI (0–32) |  |  |  |  |  |  |
| 0 | *reference* | - | *reference* | - | - | *reference* |
| 1 | 1.19 (0.57–2.51) ^3^ | - | 1.51 (0.65–3.50) | - | - | 0.54 (0.20–1.45) |
| ≥2 | 2.30 (0.90–5.91) ^3^ | - | 1.99 (0.60–6.61) ^3^ | - | - | 1.45 (0.43–4.94) ^3^ |
| RADAI score (0-48) | **1.11 (1.06–1.15) ^2,3^** | **1.15 (1.07–1.23) ^3^** | **1.20 (1.12–1.28) ^3^** | NA | NA | NA |
| TJC (0–68) | NA | NA | NA | **1.12 (1.05–1.19) ^3^** | 1.04 (0.98–1.10) ^3^ | 1.04 (0.99–1.09) ^3^ |
| SJC (0–66) | NA | NA | NA | - | - | **-** |
| Joint nodes hands | NA | NA | NA | - | - | - |
| CRP (mg/mL) | NA | NA | NA | - | - | - |
| MFI (20–100) | **1.03 (1.01–1.06) ^2,3^** | - | - | - | 1.03 (0.99–1.07) ^2^ | **1.05 (1.02–1.07)** **^2,3^** |
| VAS pain (0–10) | **1.18 (1.02–1.37) ^3^** | **1.24 (1.03–1.51) ^3^** | - | - | - | - |
| ^1^ Confounding variable in the association between age and the outcome measure (∆β_age_≥10%); ^2^ Confounding variable in the association between group and the outcome measure (∆β_group_≥10%); ^3^ Associated with the outcome measure (*p*<0.05)  **HAQ**-**DI** Health Assessment Questionnaire Disability Index; **OR** Odds Ratio; **RA** Rheumatoid Arthritis; **NA** Not Available/Applicable; **BMI** Body Mass Index; **CCI** Charlson Comorbidity Index; **RADAI** Rheumatoid Arthritis Disease Activity Index; **TJC** Tender Joint Count; **SJC** Swollen Joint Count; **CRP** C-Reactive Protein**; MFI** Multidimensional Fatigue Inventory; **VAS** Visual Analogue Scale | | | | | | |


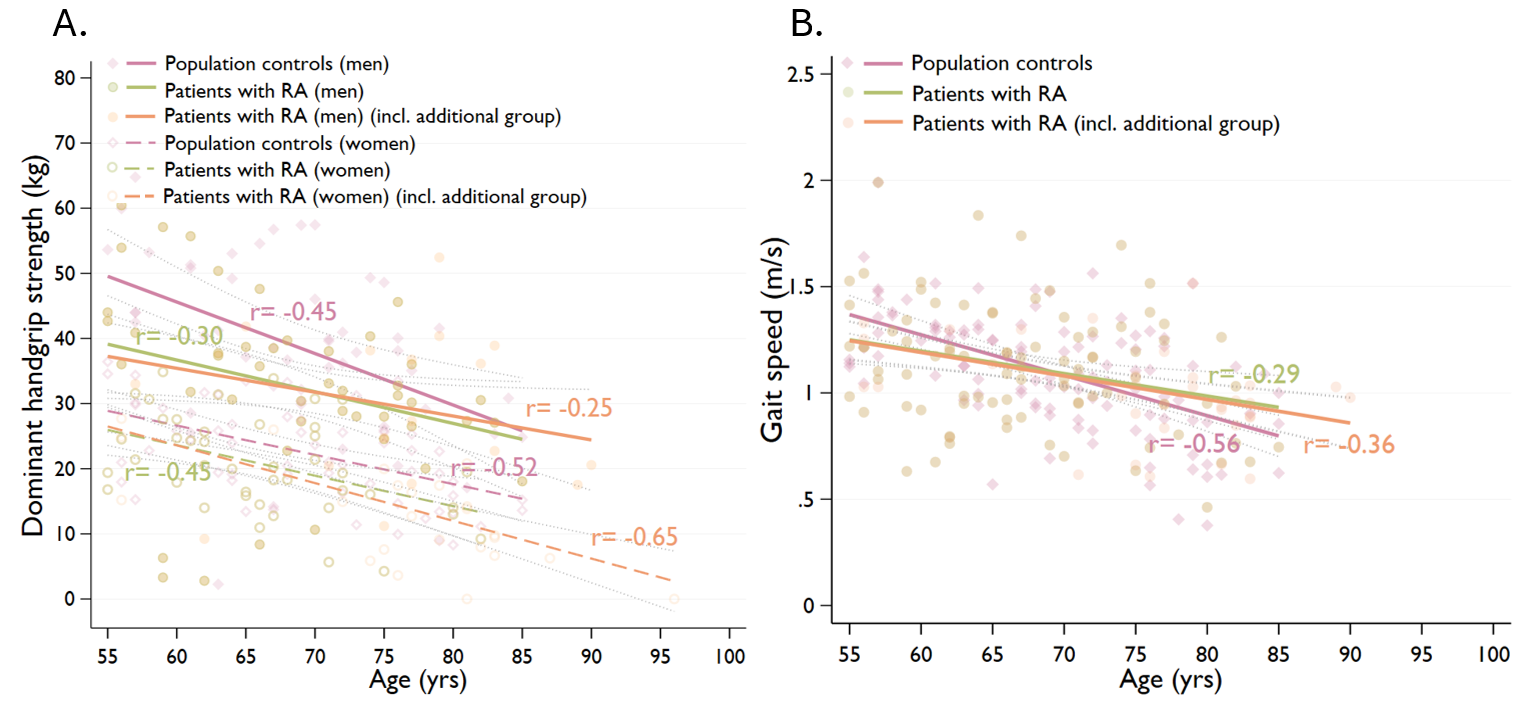


**Supplementary** **Figure S1A-B. Sensitivity analyses of dominant handgrip strength and gait speed by age, stratified for population controls (◇), patients with RA (○) and patients with RA including the additional RA group aged ≥70 years (○)**A) Observed dominant handgrip strength (kg) (controls: n_men_=48, r_men_= -0.45, n_women_=60, r_women_= -0.52; RA: n_men_=45, r_men_= -0.30, n_women_=43, r_women_= -0.45; RA incl. additional RA group aged ≥70 years: n_men_=62, r_men_=-0.25, n_women_=67, r_women_=-0.65), B) Gait speed (m/s) (controls: n=107, r= -0.56; RA: n=86, r= -0.29; RA incl. additional RA group aged ≥70 years: n=119, r= -0.36)
Abbreviations: **RA** Rheumatoid Arthritis

| Supplementary Table S4. Sensitivity analysis: Associations between age, group (RA vs. controls) and dominant handgrip strength and gait speed adding the additional RA group aged **≥70** years – multivariable linear and logistic regression | | | | |
| --- | --- | --- | --- | --- |
|  | **+ Additional RA group aged ≥70 years** | **+ Additional RA group aged ≥70 years** | **+ Additional RA group aged ≥70 years** | **+ Additional RA group aged ≥70 years** |
|  | **Dominant handgrip  strength (kg)** β (95% CI)  n=214 | **Gait speed (m/s)** β (95% CI) n=211 | **Dominant handgrip  strength (kg) Fulfil Fried criteria vs.**  **not fulfil Fried criteria** OR (95% CI) n=214 | **Gait speed (m/s) <0.8 vs. ≥0.8 s** OR (95% CI) n=212 |
| Age, *years* | **-0.3 (-0.5 to -0.2)** | **0.01 (0.01 to 0.02)** | **1.12 (1.06–1.17)** | **1.12 (1.06–1.18)** |
| Group, *patient with RA* | **-4.7 (-7.2 to -2.1)** | **0.11 (0.005 to 0.21)** | **2.92 (1.42–6.01)** | **2.77 (1.03–7.45)** |
| Sex, *women* | **-11.2 (-14.7 to -7.8)** | -0.004 (-0.13–0.13) | 0.80 (0.30–2.10) | 1.48 (0.62–3.53) |
| Height, *cm* | **0.32 (0.15–0.50) ^3^** | -0.004 (-0.011–0.003) ^1^ | 0.95 (0.90–1.00) ^3^ | - |
| Educational level |  |  |  |  |
| Low | - | - | *reference* | - |
| Middle | - | - | 0.51 (0.20–1.32) | - |
| High | - | - | **0.25 (0.10–0.66) ^3^** | - |
| Smoking status |  |  |  |  |
| No | - | *reference* | - | *reference* |
| Former | - | **-0.13 (-0.23 to -0.04) ^2,3^** | - | **0.31 (0.12–0.79) ^3^** |
| Current | - | -0.05 (-0.24–0.14) ^2^ | **^-^** | 0.81 (0.14–4.58) |
| BMI, *kg/m^2^* | - | - | - | - |
| CCI (0–32) |  |  |  |  |
| 0 | - | - | - | - |
| 1 | - | - | - | - |
| ≥2 | - | - | - | - |
| RADAI score (0-48) | NA | NA | NA | NA |
| TJC (0–68) | **-0.3 (-0.4 to -0.1) ^3^** | - | **1.10 (1.04–1.16) ^3^** | - |
| SJC (0–66) | - | - | - | - |
| Joint nodes hands | - | -0.008 (-0.018–0.003) ^2^ | - | 0.94 (0.85–1.04) ^2^ |
| CRP (mg/mL) | **-0.1 (-0.2 to -0.01) ^2,3^** | - | - | - |
| MFI (20–100) | NA | NA | NA | NA |
| VAS pain (0–10) | - | 0.003 (-0.016–0.021) ^2^ | - |  |
| ^1^ Confounding variable in the association between age and the outcome measure (∆β_age_≥10%); ^2^ Confounding variable in the association between group and the outcome measure (∆β_group_≥10%); ^3^ Associated with the outcome measure (*p*<0.05)  **RA** Rheumatoid Arthritis; **BMI** Body Mass Index; **CCI** Charlson Comorbidity Index; **RADAI** Rheumatoid Arthritis Disease Activity Index; **NA** Not Available/Applicable; **TJC** Tender Joint Count; **SJC** Swollen Joint Count; **CRP** C-Reactive Protein**; MFI** Multidimensional Fatigue Inventory; **VAS** Visual Analogue Scale | | | | |

| Supplementary Table S5. Sensitivity analysis: Associations between age, group (RA vs. controls) and HAQ-DI and Cochin hand function scale, adjusted for sociodemographic and clinical factors, in the subgroup with clinical assessments – multivariable linear and ZINB regression | | | |
| --- | --- | --- | --- |
|  | **Linear regression HAQ-DI ^1^** n=182 | **ZINB regression Cochin hand function scale ^1^** n=182 | |
|  | β (95% CI) | Logit model ^2^ OR (95% CI) | Count model ^3^ IRR (95% CI) |
| Age, *years* | 0.01 (0.01–0.02) | - | 1.02 (1.00–1.05) |
| Group, *patient with RA* | 0.12 (-0.02–0.26) | 0.33 (0.09–1.27) | 1.66 (1.05–2.63) |
| Sex, *women* | 0.25 (0.12–0.38) | 0.82 (0.22–3.04) | 1.58 (1.03–2.41) |
| ^1^ **HAQ-DI:** Additionally adjusted for BMI, CCI, RADAI and MFI; **Cochin:** Educational level, CCI, RADAI, MFI, VAS general pain; ^2^ Represents how independent variables are related to the likelihood of reporting no hand disability (Cochin score=0). OR<1 means someone is less likely to report no disability; ^3^ Represents how independent variables affect the Cochin score, among those who report any hand disability (score>0). Incidence Rate Ratio (IRR) indicates the change (%) in score  **HAQ-DI** Health Assessment Questionnaire Disability Index; **ZINB** Zero-Inflated Negative Binomial; **RA** Rheumatoid Arthritis; **BMI** Body Mass Index; **CCI** Charlson Comorbidity Index; **RADAI** Rheumatoid Arthritis Disease Activity Index; **MFI** Multidimensional Fatigue Inventory; **VAS** Visual Analogue Scale | | | |

**References supplementary material**

[1] Sokka T, Häkkinen A, Krishnan E, Hannonen P. Similar prediction of mortality by the health assessment questionnaire in patients with rheumatoid arthritis and the general population. Ann Rheum Dis. 2004 May;63(5):494-7. doi: 10.1136/ard.2003.009530. PMID: 15082478; PMID: 1754988.

[2] Wolfe F, Hawley DJ. The longterm outcomes of rheumatoid arthritis: Work disability: a prospective 18 year study of 823 patients. J Rheumatol. 1998 Nov;25(11):2108-17. PMID: 9818651.

[3] Poiraudeau S, Lefevre-Colau MM, Fermanian J, Revel M. The ability of the Cochin rheumatoid arthritis hand functional scale to detect change during the course of disease. Arthritis Care Res. 2000 Oct;13(5):296-303. doi: 10.1002/1529-0131(200010)13:5<296::aid-anr9>3.0.co;2-f. PMID: 14635299.

[4] Sferra da Silva G, de Almeida Lourenço M, de Assis MR. Hand strength in patients with RA correlates strongly with function but not with activity of disease. Adv Rheumatol. 2018 Aug 3;58(1):20. doi: 10.1186/s42358-018-0020-1. PMID: 30657069.

[5] Lefevre-Colau MM, Poiraudeau S, Fermanian J, Etchepare F, Alnot JY, Le Viet D, Leclercq C, Oberlin C, Bargy F, Revel M. Responsiveness of the Cochin rheumatoid hand disability scale after surgery. Rheumatology (Oxford). 2001 Aug;40(8):843-50. doi: 10.1093/rheumatology/40.8.843. PMID: 11511751.

[6] Lee SH, Gong HS. Measurement and Interpretation of Handgrip Strength for Research on Sarcopenia and Osteoporosis. J Bone Metab. 2020 May;27(2):85-96. doi: 10.11005/jbm.2020.27.2.85. Epub 2020 May 31. PMID: 32572369; PMCID: PMC7297622.

[7] Rijk JM, Roos PR, Deckx L, van den Akker M, Buntinx F. Prognostic value of handgrip strength in people aged 60 years and older: A systematic review and meta-analysis. Geriatr Gerontol Int. 2016 Jan;16(1):5-20. doi: 10.1111/ggi.12508. Epub 2015 May 28. PMID: 26016893.

[8] Abellan van Kan G, Rolland Y, Andrieu S, Bauer J, Beauchet O, Bonnefoy M, Cesari M, Donini LM, Gillette Guyonnet S, Inzitari M, Nourhashemi F, Onder G, Ritz P, Salva A, Visser M, Vellas B. Gait speed at usual pace as a predictor of adverse outcomes in community-dwelling older people an International Academy on Nutrition and Aging (IANA) Task Force. J Nutr Health Aging. 2009 Dec;13(10):881-9. doi: 10.1007/s12603-009-0246-z. PMID: 19924348.

[9] Hornyak, V., VanSwearingen, J.M., and Brach, J.S. "Measurement of gait speed." *Topics in geriatric rehabilitation* 28.1 (2012): 27-32.

[10] Makizako, H., Shimada, H., Doi, T., Tsutsumimoto, K., Nakakubo, S., Hotta, R., & Suzuki, T. (2017). Predictive cutoff values of the five-times sit-to-stand test and the timed “up & go” test for disability incidence in older people dwelling in the community. *Physical therapy*, *97*(4), 417-424.

[11] Hughes, M. A., Myers, B. S., & Schenkman, M. L. (1996). The role of strength in rising from a chair in the functionally impaired elderly. *Journal of biomechanics*, *29*(12), 1509-1513.

[12] Lord, S. R., Murray, S. M., Chapman, K., Munro, B., & Tiedemann, A. (2002). Sit-to-stand performance depends on sensation, speed, balance, and psychological status in addition to strength in older people. *The Journals of Gerontology Series A: Biological Sciences and Medical Sciences*, *57*(8), M539-M543.

[13] Goldberg, A., Chavis, M., Watkins, J., & Wilson, T. (2012). The five-times-sit-to-stand test: validity, reliability and detectable change in older females. *Aging clinical and experimental research*, *24*(4), 339-344.
